# Measuring Soluble ICAM-1 in African Populations

**DOI:** 10.1371/journal.pone.0108956

**Published:** 2014-10-07

**Authors:** Abdirahman I. Abdi, Michelle Muthui, Esther Kiragu, Peter C. Bull

**Affiliations:** 1 KEMRI-Wellcome Trust Research Programme, Kilifi, Kenya; 2 Nuffield Department of Clinical Medicine, John Radcliffe Hospital, University of Oxford, Oxford, United Kingdom; 3 Department of Biochemistry and Chemistry, Pwani University, Kilifi, Kenya; Université Pierre et Marie Curie, France

## Abstract

The level of plasma soluble ICAM-1 (sICAM-1) has been associated with the pathogenesis of several diseases. Previously, a commercial antibody was reported not to recognize an ICAM-1 allele known as ICAM-1^kilifi^ prevalent among African populations. However, that study was based on 19 samples from African Americans of whom 13 had the wild type allele, five heterozygotes and one homozygote. Here, we compare plasma sICAM-1 measures using three different commercial antibodies in samples from Kenyan children genotyped for ICAM-1^kilifi^ allele. We show that two of these antibodies have some degree of deficiency in detecting the ICAM-1^kilifi^ allele. Consideration of the antibody used to measure sICAM-1 is important as up to 30% of the populations in Africa harbour this allele.

## Introduction

Adhesion molecules expressed on vascular endothelial cells in response to inflammation are crucial for the pathogenesis of infectious (reviewed in [1]) and non-infectious diseases [2]. One well studied molecule is the intercellular adhesion molecule-1 (ICAM-1) expressed on endothelial cells where its expression is amplified by pro-inflammatory cytokines [3]. ICAM-1 plays a role in recruitment of leukocytes to the vascular wall in response to inflammation [4]. In malaria, it plays a role in the sequestration of infected erythrocytes in the organs [5], leading to blockage of blood vessels and coma. ICAM-1 is also a receptor for Rhinovirus [6].

ICAM-1 protein is found in both membrane-bound and soluble forms. The soluble form is thought to arise from active cleavage of the endothelial membrane-bound form by a zinc dependent metalloprotease [7]. Soluble levels of ICAM-1 (sICAM-1) in plasma have been associated with coronary heart disease and other vascular diseases [2]. In malaria, sepsis, and other infectious diseases, increasing plasma sICAM-1 has been associated with severity of disease (reviewed in [1]).

Polymorphisms within the ICAM1 coding gene have been shown to influence the level of plasma sICAM-1 [8]. Of relevance to this report is a single nucleotide point mutation (SNP) at the rs5491 first reported by Fernandez-Reyes et al [9], who sequenced the coding region from Kenyan children. This SNP leads to a non-synonymous mutation that brings about a change from lysine to methionine (K29M) and this variant is referred to as ICAM-1^kilifi^. The frequency of this allele is around 20–30% in many African populations [9]–[12] and is rare among Caucasian populations [13].

A study by Register et al [13] first reported that a monoclonal antibody from R&D Systems (BBE1B) does not recognize ICAM-1^kilifi^. Another study using the same R&D Systems antibody found that the levels of sICAM-1 reduces by 50% in the heterozygotes (K29/M29), while the homozygotes (M29/M29) have levels below detection [8]. To examine whether the low sICAM-1 levels observed with the R&D monoclonal antibody is due to inability of the antibody to recognize the ICAM-1^kilifi^ allele rather than unavailability of the protein, Register et al [13] used an alternative monoclonal α-sICAM-1 (BMS201INST; Bender MedSystems) in ELISA. With this alternative antibody, sICAM-1 was recognized to a greater extent in plasma samples from both heterozygotes and homozygotes suggesting that the almost background sICAM-1 levels observed in the homozygotes (M29/M29) with the R&D antibody (BBE1B) is likely due to its inability to detect the ICAM-1^kilifi^ allele. However, the sample size in Register’s study was small where the homozygotes were represented by only one sample and therefore we sought to confirm this finding in a larger sample size.

It was also reported, using another antibody from R&D systems (catno. DY720), that the plasma levels of sICAM-1 are not affected by the ICAM-1^kilifi^ allele [14], though no data was given. Since the original R&D antibody BBE1B showing marked variation between alleles is no longer marketed under that name, we performed a comparative study of plasma sICAM-1 measures using two antibodies from R&D (DCD540 and DY720) and the antibody from Bender MedSystems (BMS201MST) in a group of Kenyan children genotyped for ICAM-1^kilifi^ allele. We show that the antibody in the kit DCD540 is hardly able to detect the ICAM-1^kilifi^ allele whilst the BMS201MST antibody has reduced ability to detect this allele. We confirm that DY720 antibody is able to detect sICAM-1 equally for both alleles.

## Methods

### Ethics statement

Ethical approval was obtained from Kenya Medical Research Institute (KEMRI) Ethical Review Committee (SSC 1131), and written informed consent was obtained from parents/Guardians of the study participants.

### ICAM-1 genotyping and sicam1 ELISA

Samples used in this study are from children presenting with malaria to Kilifi county hospital, a rural town located at the coast of Kenya. The children were recruited between August 2003 and September 2007 [15]. We used published methods for host genotyping of the K29M polymorphism of ICAM1 [16] and primers described in [10].

Two ELISA kits from R&D were used; catalog numbers DCD540 and DY720 and one from Bender MedSystems; catalog number BMS201MST to measure plasma sICAM-1 (note, BMS201MST and BMS201INST contain the same antibody). The measurements were performed according to the manufacturer’s protocols. The BBE1B antibody that was previously used in Register et al study is no longer marketed under this name. The DCD540 and BMS201MST kits contain monoclonal antibodies. No information about whether the antibodies in the DY720 kit are monoclonal or polyclonal is available in the product’s datasheet. The manufacturer of the DCD540 kit states the inability of the antibody in the kit to recognize ICAM-1^kilifi^ allele in the product datasheet.

Figures were generated using GraphPad Prism software version 5. We tested for trend across the genotypes by Cuzick’s test for trend [17] using Stata software version 12. The Cuzick test was used because it includes a correction for tied ranks.

## Results and Discussion

**Figure 1:**
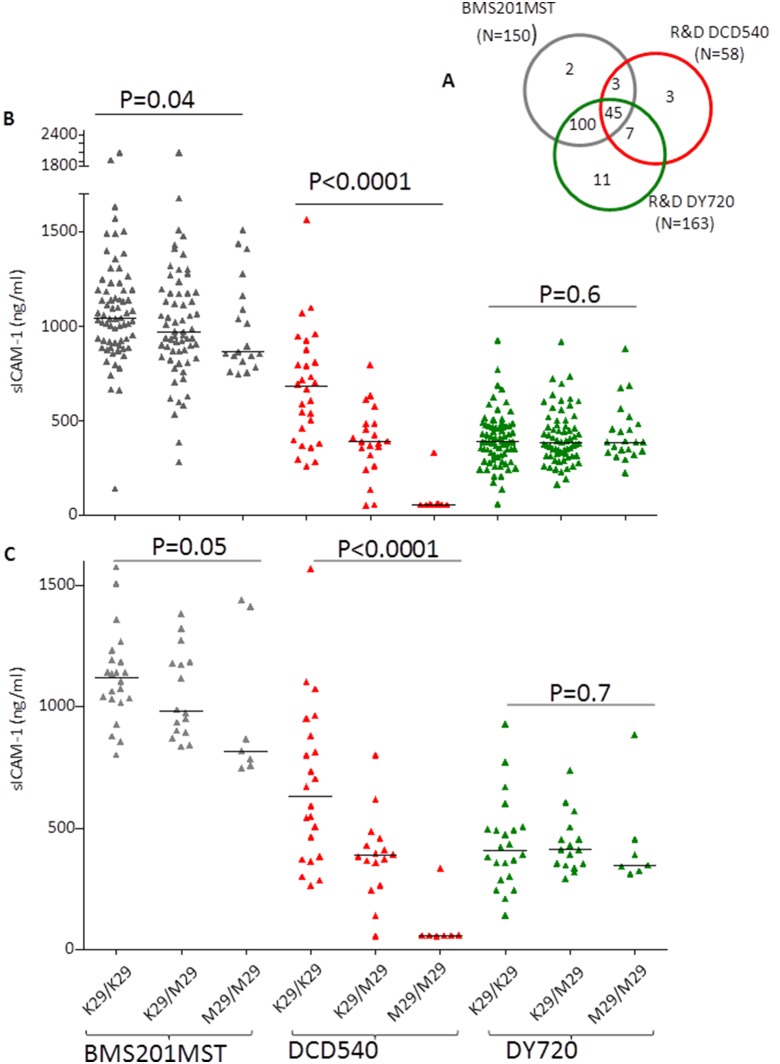
Plasma sICAM-1 in relation to ICAM-1^kilifi^ genotypes. A. Venn diagram showing the samples used in this study We measured sICAM-1 in 163, 150, and 58 samples using DY720, BMS201MST, and DCD540 ELISA kits. These samples were genotyped from ICAM-1^kilifi^ mutation. Of these, 145 samples have been measured with both BMS201MST and R&D DY720, 52 with R&D DY720 and R&D DCD540, 48 with BMS201MST and R&D DCD540, and 45 with all the three kits. B. Plasma sICAM1 obtained with three different kits relative to ICAM-1^kilifi^ genotypes C. Plasma sICAM-1 in a subset of 45 samples where a measure was obtained with each of the three antibodies used. Measures of plasma sICAM-1 obtained with the BMS201MST, R&D (DCD540) and R&D (DY720) antibodies are depicted in black, red and green triangles respectively. The horizontal bar indicates the median sICAM-1 value. P value was calculated using Cuzick’s test for trend.

Plasma sICAM-1 for 58, 163 and 150 samples genotyped for ICAM-1 K29M polymorphism were obtained using the DCD540, DY720 and BMS201MST ELISA kits respectively as shown in figure 1A. Of these, 45 samples had a measure of sICAM-1 with each of the three kits (Fig. 1A).

Initially, we used the DCD540 antibody to measure sICAM-1 in 58 plasma samples genotyped for ICAM-1^kilifi^. We found that the plasma sICAM-1 levels decrease from wild-type, ICAM-1^kilifi^ heterozygoyes and homozygotes in that order (Figure 1B). This decreasing trend in the levels of sICAM-1 was statistically significant (P<0.0001, Cuzick’s test for trend). This result is similar to that previously reported with the R&D (BBE1B) monoclonal antibody [13].

The Register et al [13] study used another Kit (BMS201INST) to demonstrate that expression of sICAM1 was not altered by the ICAM-1^kilifi^ allele [13]. However this was difficult to interpret, as the sample size was small. We therefore measured sICAM-1 in 150 samples genotyped for ICAM-1^kilifi^ allele using the BMS201MST antibody. With this antibody, although detection of sICAM-1 improved in both heterozygotes and homozygotes, we still observed a significant decreasing trend in the levels when moving from 0 to 1 to 2 copies of the ICAM-1^kilifi^ allele (Fig. 1B; P = 0.04, Cuzick’s test for trend) suggesting a deficiency in the ability of BMS201MST to detect ICAM-1^kilifi^ allele.

Finally, as a second alternative to BMS201MST we measured plasma sICAM-1 in 163 samples genotyped for ICAM-1^kilifi^ allele using the DY720 antibody from R&D Systems. With this antibody, the median levels were the same in the different ICAM-1^kilifi^ genotypes (Fig. 1B) confirming that the antibody in this kit is not affected by the presence of the ICAM-1^kilifi^ allele [14]. To directly compare the BMS201MST, and DY720, we compared their performance in the 145 samples with sICAM-1 measures with both antibodies. Again, while the sICAM-l levels obtained with BMS201MST showed a significant decreasing trend from wild type to heterozygotes to homozygotes (N = 145, P = 0.03, Cuzick’s test for trend), the measures obtained with DY720 showed no difference (N = 145, P = 1, Cuzick’s test for trend). A similar trend was observed with 45 samples for which we had a measure of plasma sICAM-1 against all the three antibodies (Fig. 1C).

The result obtained with DCD540 antibody used in this study is largely consistent with that described in Register et al [13] using the BBE1B monoclonal antibody. Our result also suggests that the monoclonal antibody in the BMS201MST kit has some deficiency in detecting sICAM-1^kilifi^ allele (Fig. 1B). Of the three antibodies we tested, the DY720 antibody of the R&D Systems seems to be the ideal for measuring sICAM-1 levels in samples from African individuals and the DCD540 to be the least effective.

As the frequency of ICAM-1^kilifi^ allele is reported to be as high as 30% in some parts of Africa, we sought to know the number of studies carried in Africa that measured sICAM-1 and what kind of antibodies they used (summarised in table 1). We found that the majority of the studies were done in the context of malaria and used the DY720 antibody [18]–[21]. Other studies stated that they used a kit from R&D without specifying [22], [23].

**Table 1:** Published work done in Africa that measure sICAM-1.

Article	Kit used to measure sICAM-1
Bellamy R et al, Trans R Soc Trop Med Hyg 1998 [10]	R&D DY720
Tchinda VH et al Acta Trop 2007 [18]	R&D DY720
Conroy AL et al plos One 2010 [19]	R&D DY720
Cserti-Gazdewich CM et al, Malar J. 2010 [20]	R&D DY720
Adukpo, S et al, Plos One 2013 [21]	R&D DY720
Djoba Siawaya JF et al, J infect 2008 [22]	R&D but not specified
Park GS et al, J Pediatric Infect Dis Soc 2012 [23]	R&D but not specified
Mita-Mendoza, NK et al Plos One 2013 [25]	R&D LAD000 (Human Adhesion Molecule MultiAnalyte Profiling Base Kit)
El-Deek S.E et al Med Princ Pract 2013 [26]	Bender MedSystem GmbH, Campus Vienna Biocenter

Since, in a large study, the ICAM-1^kilifi^ polymorphism was shown not to be associated with severe malaria [12], it is unlikely that this would have altered the conclusions in studies with severe malaria (and this is supported by a personal communication from Parks GS & John CC). Nevertheless, the high prevalence of this mutation among African populations raises the possibility that it is selected for by other conditions prevalent in Africa including other infectious diseases. This is supported by a previous study that found an association between ICAM-1^kilifi^ allele and reduced incidence of non-malarial febrile illness in Kenyan children [24]. Therefore, consideration of the effect of ICAM-1^kilifi^ allele is important because of its impact on the performance of some of the commercial ELISA kits used to measure sICAM-1.
